# PRINTO scholarships: the Italian experience

**DOI:** 10.1186/1546-0096-5-18

**Published:** 2007-09-27

**Authors:** Pablo Garcia Munitis

**Affiliations:** 1 9N°140 CP 1900. La Plata, Argentina

## Abstract

The increasing availability of the internet allows physicians to access actualized medical information quickly and easily, but it is not comparable with the possibility of working in a well known international medical centre.

International collaboration (scholarships, courses and research), such as the PRINTO alpha project, allows professionals not only to increase and share scientific knowledge and experiences but also to integrate into a working team in a foreign country which leads to an understanding among cultures.

PRINTO has set up a scientific and technical collaborative research network in Paediatric Rheumatology for Latin American physicians.

Having been a fellow of the alpha project at "Gianina Gaslini Hospital" in Genova, Italy, I read the article in your journal written by Nicolino Ruperto and Alberto Martini [1] and I thought that the paragraphs referring to "Research training in Paediatric rheumatology" could have been treated in more detail, considering both the scientific and human aspects of the Alpha Project.

The purpose of the Paediatric Rheumatology International trial organization (PRINTO) is to foster, facilitate and conduct high quality research in the field of paediatric rheumatology (PRINTO bylaws) [2]. This program was set up on the basis that paediatric rheumatic diseases are rare, consequently, qualified physicians are needed to conduct multicenter collaborative studies on an international scale, to obtain clinically and statistically valid results.

Twenty four fellowships were available for Latin American professionals. Those fellows who participated in Genova Alpha Project not only succeeded in paediatric rheumatic research but also went through an invaluable human and cultural experience [2].

The scientific program included:

1. Clinical activities

a. Daily inpatients visit.

b. Weekly meeting with Prof. Martini, director of the unit, and the staff physicians.

c. Acquisition of techniques for intra-articular injections in the main joints, either blinded or ultrasound-driven.

2. Courses

a. Course in Clinical trials and applied statistics, held by Dr Nicolino Ruperto MD, MPH.

b. Course in outcome assessment for the paediatric rheumatic diseases, held by Dr Angelo Ravelli MD.

c. Course in Basic and Intermediate Biostatistics and Epidemiology, held by Dr Angela Pistorio, MD, PhD.

3. Research activities with active participation in research projects, some of them published in different medical journals [3,4].

Furthermore we participated in a Language and Culture course, where we could learn the Italian language and lots of things concerning Italian culture and history.

What I do want also to emphasize is the host's cordiality, dedication and professionalism, and their positive disposition to integrate the fellows to a working team in a foreign country which, no doubt, leads to an understanding among nations.

My most sincere gratitude to all of them and a strong wish that this experience could be repeated.
